# Modulatory Effect of Fermented Papaya Extracts on Mammary Gland Hyperplasia Induced by Estrogen and Progestin in Female Rats

**DOI:** 10.1155/2017/8235069

**Published:** 2017-11-20

**Authors:** Zhenqiang You, Junying Sun, Feng Xie, Zhiqin Chen, Sheng Zhang, Hao Chen, Fang Liu, Lili Li, Guocan Chen, Yisheng Song, Yaoxian Xuan, Gaoli Zheng, Yanfei Xin

**Affiliations:** ^1^State Key Laboratory of Safety Evaluation for New Drugs, Zhejiang Academy of Medical Sciences, Hangzhou, Zhejiang, China; ^2^Institute of Animal Health, Guangdong Academy of Agricultural Sciences, Guangzhou, Guangdong, China; ^3^Collaborative Innovation Center of Yangtze River Delta Region Green Pharmaceuticals, Zhejiang University of Technology, Hangzhou, Zhejiang, China

## Abstract

Fermented papaya extracts (FPEs) are obtained by fermentation of papaya by *Aspergillus oryzae* and yeasts. In this study, we investigated the protective effects of FPEs on mammary gland hyperplasia induced by estrogen and progestogen. Rats were randomly divided into 6 groups, including a control group, an FPE-alone group, a model group, and three FPE treatment groups (each receiving 30, 15, or 5 ml/kg FPEs). Severe mammary gland hyperplasia was induced upon estradiol benzoate and progestin administration. FPEs could improve the pathological features of the animal model and reduce estrogen levels in the serum. Analysis of oxidant indices revealed that FPEs could increase superoxide dismutase (SOD) and glutathione peroxidase (GSH-Px) activities, decrease malondialdehyde (MDA) level in the mammary glands and serum of the animal models, and decrease the proportion of cells positive for the oxidative DNA damage marker 8-oxo-dG in the mammary glands. Additionally, estradiol benzoate and progestin altered the levels of serum biochemical compounds such as aspartate transaminase (AST), total bilirubin (TBIL), and alanine transaminase (ALT), as well as hepatic oxidant indices such as SOD, GSH-Px, MDA, and 8-oxo-2′-deoxyguanosine (8-oxo-dG). These indices reverted to normal levels upon oral administration of a high dose of FPEs. Taken together, our results indicate that FPEs can protect the mammary glands and other visceral organs from oxidative damage.

## 1. Introduction

Fermented fruit and vegetable juices are popular functional foods in China. They contain large amounts of probiotics, amino acids, carbohydrates, and functional enzymes, which can be easily digested. In addition, fermented fruit and vegetable juice exhibits antioxidant, antitumor, antidiabetic, and anti-inflammatory activities [1, 2]. Papaya is used in traditional medicine because of its antioxidant, antitumor, and immune regulatory effects [3–6]; these effects could be due to the high content of *α*-tocopherol, flavonoid, benzyl isothiocyanate, lycopene, and papain in papaya [7–10]. Fermented papaya is used in medical treatment and has been developed as a commercial functional food in Japan, United States, and several European countries [11]. Studies have shown that fermented papaya products (FPP) protect hemocytes from oxidative damage, prevent complications of aging, and cure Alzheimer's disease (AD) [12–14]. Due to its antioxidant activity, FPP exhibits potential for tumor treatment, immune regulation, and wound healing in diabetics [4, 6, 15]. Fermented papaya extracts (FPEs) are the fermentation liquor extracted from papaya through long-term fermentation by *Aspergillus* and yeast; they are commercially available in the form of tablets and powder. Clinically, FPEs are applied as functional food to regulate endocrine disorders and antioxidant activity, as well as to improve immune and anti-inflammatory responses.

Mammary gland hyperplasia is a common noninflammatory and nontumor disease that occurs in women of child-bearing age. This condition accounts for 75% of all breast diseases [16, 17]. Women suffering from mammary gland hyperplasia exhibits two to three times higher risk of breast cancer than the general population. The risk is five to eight times higher among patients with atypical hyperplasia [18, 19]. The occurrence of mammary gland hyperplasia is directly related to endocrine disorders, which are mainly caused by imbalance of estrogen and progestin [20]. Hyperplasia of mammary epithelial cells can also be induced by cholesterol and its oxidative product cholesterol epoxide [21, 22]. In the present study, animal models of mammary gland hyperplasia were established using estradiol benzoate and progestin based on the underlying mechanism of mammary gland hyperplasia. Using these models, the protective effect, functions, and mechanism of FPEs in estrogen-induced mammary gland hyperplasia were investigated.

## 2. Materials and Methods

### 2.1. FPEs and Drugs

Immature papaya (half a month from maturity) were peeled and cut into 2 mm slices after removing the seeds. The prepared papaya was fermented for 3 months through *Aspergillus oryzae* (isolated from millet catsup and stored at our laboratory), and then fermented for 3 months using yeasts (*Saccharomyces cerevisiae*, provided by Desheng Biotechnology Co. Ltd., Zhejiang, China) with 5% glucose at 24–28°C. The FPEs were obtained by squeezing and filtering after fermentation. Total flavonoids, one of the important functional constituents in FPEs, were nearly 11.65 mg/ml detected by UV method. Estradiol (2 mg/ml) and progestin (10 mg/ml) were purchased from Hangzhou Animal Pharmaceutical Factory, China.

### 2.2. Animals and Treatments

Thirty-six female SPF Sprague-Dawley (SD) rats (180–200 g, 7-8 weeks old, and at sexual maturity) were purchased from the Shanghai Slack Laboratory Animal Co. Ltd. All animals were groomed in the barrier system with a clean environment at 20°C–25°C under 50–60% humidity and with a 12 h light and 12 h dark cycle. Animal experiments were conducted in laboratories that passed the AAALAC (Association for Assessment and Accreditation of Laboratory Animal Care International) authentication. The rats were randomly divided into six groups, with each group containing six rats.

Group 1, the blank control group, received daily intramuscular injection of 0.5 ml/kg saline and were orally administered 30 ml distilled water/kg weight. Group 2, the FPE control group, was orally administered 30 ml of FPEs/kg weight at the beginning of the experiment. Group 3, the model group, received intramuscular injection of 0.5 mg (0.5 ml)/kg estradiol benzoate from day 1 to day 25 and 4 mg (0.5 ml)/kg progestin from day 26 to day 30. As treatment groups, group 4, group 5, and group 6 received estradiol benzoate and progestin the same as the model group and were orally treated with FPEs from day 1 to day 30. Group 4, treatment group I, was administered an oral dose of 30 ml of FPEs/kg. Group 5, treatment group II, was administered an oral dose of 15 ml of FPEs plus 15 ml of distilled water/kg. Group 6, treatment group III, was administered an oral dose of 5 ml of FPEs plus 25 ml of distilled water/kg.

### 2.3. Nipple Measurement

On the 31st day, the third nipple on the right side of the rats in each group was shaved. The diameter and height of the shaved nipple were measured using a Vernier caliper.

### 2.4. Sample Collection

On the 31st day, all experimental rats were anesthetized with 2.5% pentobarbital solution and killed humanely. Blood and tissue samples were collected from the mammary glands, heart, liver, spleen, lung, kidney, ovary, and uterus. To avoid interference from blood cells, the blood in the liver was cleared by injecting saline through the portal vein. Once blood clotted at room temperature, the serum was separated within 10 min by centrifugation at 3000 ×g. A portion of the serum was immediately used for biochemical detection, and the remainder was stored at −70°C for analysis of hormones and oxidant indices. Half of the collected tissue samples were stored in 10% formaldehyde for pathological and immunohistochemical (IHC) tests, and the remaining half was stored at −70°C for testing oxidant indices and 8-oxo-2′-deoxyguanosine (8-oxo-dG).

### 2.5. Sex Hormone Concentrations

The concentration of each sex hormone in the serum was tested as per standard specifications using estradiol (E2), progesterone (P), luteinizing hormone (LH), and follicle-stimulating hormone (FSH) detection kits (Beijing Kemei Biological Technology Co. Ltd.).

### 2.6. Oxidative Index Detection

Malondialdehyde (MDA) level, superoxide dismutase (SOD), and glutathione peroxidase (GSH-Px) activities in the serum, mammary glands, and livers were detected using commercially available detection kits following the manufacturer's instructions (Sigma-Aldrich).

### 2.7. Blood Biochemistry

Serum biochemical parameters including aspartate transaminase (AST), alanine transaminase (ALT), total bilirubin (TBIL), alkaline phosphatase (ALP), creatine kinase (CK), blood urea nitrogen (BUN), creatinine (Crea), total protein (TP), albumin (ALB), glucose (GLU), total cholesterol (T-CHO), triglyceride (TG), potassium (K^+^), serum sodium (Na^+^), chloride (Cl^−^), and total calcium (TCa) were analyzed using a Hitachi 7020 automatic biochemical analyzer.

### 2.8. Detection of 8-Oxo-2′-deoxyguanosine (8-oxo-dG) through Enzyme-Linked Immunosorbent Assay (ELISA) and IHC

The biomarker 8-oxo-dG was detected in the mammary glands and liver tissues through ELISA and IHC analyses. For ELISA, 50 mg of the tissues was collected. Total DNA was extracted using a genome extraction kit (Sigma-Aldrich) and quantified by colorimetric method. Approximately 300 ng of DNA was extracted from each sample. The content of 8-oxo-dG in the total DNA was determined using the EpiQuik™ 8-OHdG DNA Damage Quantification Direct Kit (Colorimetric) (EpiGentek). The results are presented as the ratio of 8-oxo-dG-positive cells.

For IHC, mammary glands were fixed in 10% formaldehyde for at least 24 h and sliced into 4 *μ*m sections after embedding in wax. After dewaxing, hydration, antigen retrieval, DNA hybridization, and sealing treatment, the slices were incubated overnight in anti-8-oxo-dG monoclonal antibody (1 : 250, Trevigen) at 4°C. Next, the slices were incubated in DyLight 594-labled goat anti-mouse IgG (1 : 250, Jackson) for 30 min. After washing, the slices were evaluated using a fluorescence microscope.

### 2.9. Pathological Mechanism Detection

Each major visceral organ was fixed in 10% formaldehyde for at least 24 h, sliced into 4 *μ*m sections after embedding with wax, and dyed with standard HE dyeing procedures. The pathological state of each visceral organ was observed by optical microscopy. Signs of pathological damage, such as lobule increase, acinar increase, mammary duct and lumen ectasia, and mammary duct and lumen secretion, were analyzed.

### 2.10. Statistical Analysis

Results are reported as mean ± SEM (standard error of mean) and were analyzed using SPSS 22.0. One-way ANOVA was conducted to compare data among more than two groups. *p* < 0.05 was considered statistically significant.

## 3. Results

### 3.1. Protective Effect of FPEs on Serum Sex Hormone Index of Rats

The regulatory effect of FPEs on sex hormone concentration in the model rats was determined by detecting serum E2, P, LH, and FSH levels. After administering the model rats with estradiol benzoate and progestin, the serum levels of these hormones increased (compared with those in the control group, *p* < 0.05). After treatment with FPEs, the levels of E2 and FSH were found to be evidently decreased (compared with those in the model group, *p* < 0.05) and dose-dependent. In addition, the level of P was significantly decreased in the larger-dose (30 ml of FPEs/kg) group, and the level of LH was obviously reduced both in the larger-dose group and the middle-dose (15 ml of FPEs/kg) group (compared with those in the model group, *p* < 0.05) (Table 1).

### 3.2. Effect of FPEs on Nipple Size of Rats

The diameter and height of the nipples of rats increased upon estradiol benzoate and progestin administration (compared with those in the control group, *p* < 0.05). However, upon treatment with FPEs, the diameter and height of their nipples improved. In the larger-dose group, the diameter and height of their nipples were significantly attenuated (compared with those in the model group, *p* < 0.05). Additionally, the middle-dose group apparently alleviated the diameter of nipples. However, the smaller-dose (5 ml of FPEs/kg) group did not show an evident modulatory role in the diameter and height of nipples (Table 1).

### 3.3. Protective Effect of FPEs on Mammary Gland Pathology in Model Animals

**Figure 1:**
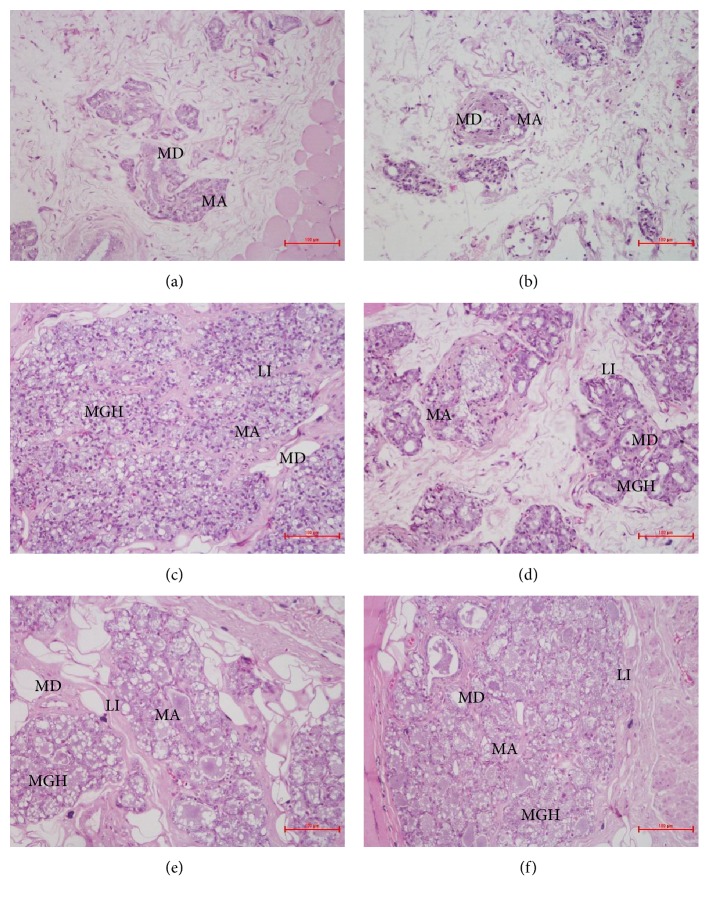
Photomicrographs showing mammary gland of rats in the control and treatment groups (200x). There was no apparent hyperplasia and no clear secretion in the mammary ducts and lumens in the control group (a) and the FPE-treatment-alone group (b). However, severe mammary gland hyperplasia (MGH), lobule increase (LI), acinar (MA) increase, mammary duct (MD) and lumen ectasia, and mammary duct and lumen secretion were observed in the estradiol benzoate and progestin treatment group (c). The pathology of the mammary glands of the model rats improved upon treatment with FPEs at 30 ml/kg (d), 15 ml/kg (e) and 5 ml/kg (f). Scale bar, 100 *μ*m.

In this study, mammary gland hyperplasia was induced by administering estradiol benzoate and progestin and treated with FPEs. Histopathological analysis showed that the lobules and acinars in the control group and group administered with FPEs alone showed no apparent hyperplasia, with few acinars and no clear secretion in the mammary ducts and lumens. By contrast, severe pathological changes, including mammary gland hyperplasia, lobule increase, acinar increase, mammary duct and lumen ectasia, and mammary duct and lumen secretion increase, were observed in the model group.  Table 2 shows the statistics for several pathological features related to mammary gland hyperplasia across the study groups. The pathology of the mammary glands in the model rats given with the larger dose and middle dose of FPEs improved, with significant changes observed in lobule number, acinar number, and mammary duct and lumen secretion. Mammary duct and lumen secretion in the treatment group administered a high dose of FPEs almost returned to the normal level. In addition, pathological improvements of mammary gland hyperplasia were found to be positively correlated with FPE dosage (Figure 1).

### 3.4. FPEs Improve Mammary Gland Hyperplasia in Model Rats via Its Antioxidant Effects

**Figure 2:**
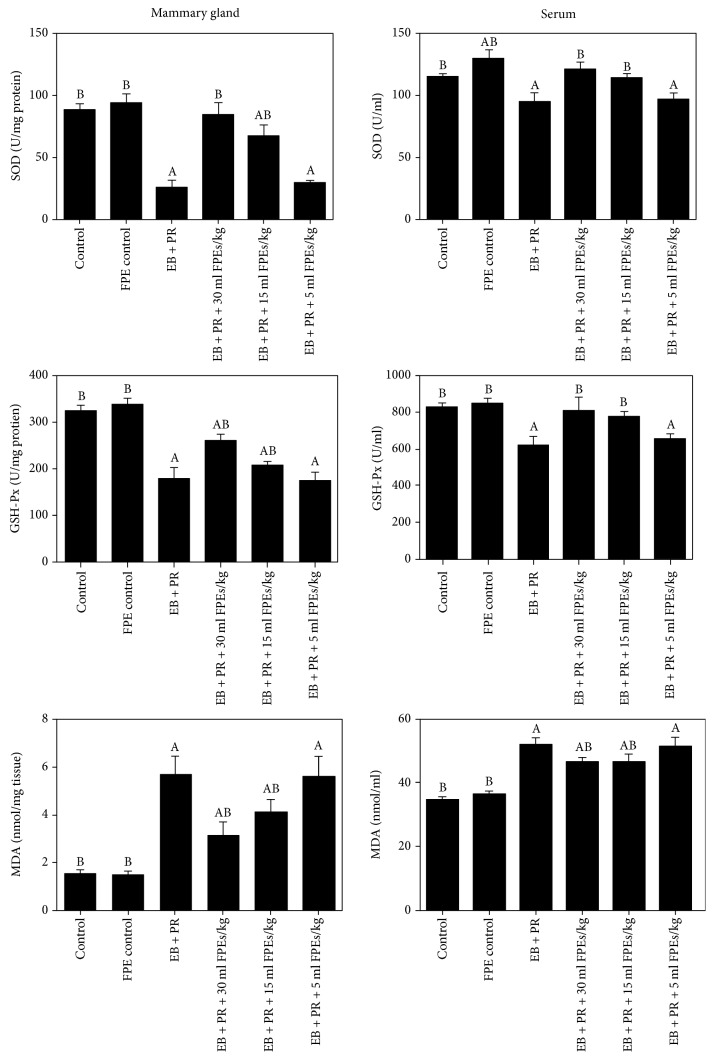
Effect of FPEs on SOD and GSH-Px activities and MDA level in the serum and mammary glands, which had changed upon treatment with estradiol benzoate and progestin. Data are presented as mean ± SEM. ^A^*p* < 0.05 compared with the control group; ^B^*p* < 0.05 compared with the EB + PR group.

Mammary gland hyperplasia induced by estradiol benzoate and progestin resulted in oxidative damage. Oxidant indices such as SOD and GSH-Px activities and MDA level in the serum and mammary glands were detected the day after mammary gland hyperplasia was induced. Therefore, we sought to evaluate the protective effect of FPEs against oxidative damage in the mammary glands. As shown in Figure 2, SOD and GSH-Px activities and MDA level in the mammary glands and serum of the model rats obviously changed compared with that in the control group (*p* < 0.05). These oxidative indices in the mammary glands and serum of the model rats also significantly improved after treatment with FPEs both in the larger-dose group and the middle-dose group (compared with those in the model group, *p* < 0.05). SOD and GSH-Px activities in the serum of model rats treated with high doses of FPEs recovered to levels observed in the control group. Moreover, SOD activity in the serum of rats treated with FPEs alone (group 2) was higher than that in the control group (*p* < 0.05). Although the smaller-dose group did not show an apparent antioxidant effect, these results indicate that FPEs can increase the antioxidant levels and inhibit the hyperplasia of mammary glands by antioxidants.

### 3.5. Blood Biochemistry

**Figure 3:**
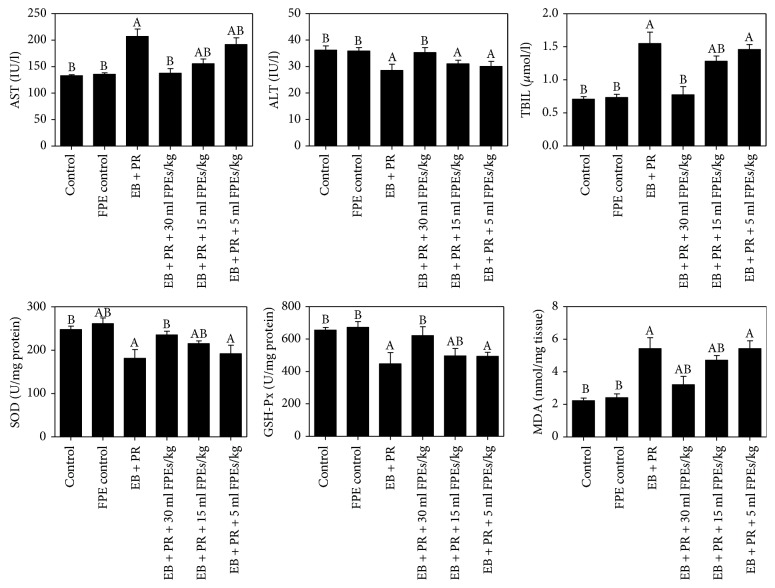
Effect of FPEs on AST, ALT, and TBIL biochemistry indices and SOD, GSH-Px, and MDA oxidative indices in the livers, which changed upon treatment with estradiol benzoate and progestin administration. Data are presented as mean ± SEM. ^A^*p* < 0.05 compared with the control group; ^B^*p* < 0.05 compared with the EB + PR group.

Each serum biochemical index was determined to investigate the damage induced by estradiol benzoate and progestin in the body and the protective effect of FPEs. The AST and TBIL indices in the model rats obviously increased, and the ALT index obviously decreased in the model rats (compared with those in the control group, *p* < 0.05). Compared with those in the model group, the AST, TBIL, and ALT indices in the larger-dose FPE treatment group were obviously improved and recovered to the levels of the control group (*p* < 0.05). Furthermore, FPEs showed a dose-dependent effect on AST index. The TBIL index was also evidently reduced in the middle-dose group. However, the TBIL and ALT indices in the smaller-dose group were not significantly changed (compared with those in the model group, *p* > 0.05) (Figure 3).

### 3.6. FPEs Inhibit Liver Damage in Model Rats via Their Antioxidant Effects

Estradiol benzoate and progestin can induce mammary gland hyperplasia and change AST, TBIL, and ALT, which suggested that estradiol benzoate and progestin could induce damage to other visceral organs. Pathological examination of the major visceral organs did not reveal any obvious pathological change in the visceral organs, except for the liver, which underwent inflammatory infiltration. Mammary gland hyperplasia is inhibited by FPEs mainly via antioxidants. The SOD and GSH-Px activities and MDA levels in the liver were examined to determine whether liver damage induced by estradiol benzoate and progestin was caused by oxidative stress and whether the liver was protected by FPEs via antioxidants. The liver was completely cleaned with saline to avoid interference from blood. Results showed that SOD and GSH-Px activities were obviously decreased, and the MDA level was increased in the model group (compared with those in the control group, *p* < 0.05). These oxidative indices in the liver tissues of the model rats treated with FPEs were obviously restored both in the larger-dose group and the middle-dose group. The SOD and GSH-Px activities in the larger-dose group were increased to the same level as the control group (compared with those in the model group, *p* < 0.05, and compared with those in the control group, *p* > 0.05). However, the smaller-dose group did not show a significant protective effect. In addition, the SOD activity in rats orally administered FPEs alone was higher than that in the control group (*p* < 0.05). Thus, our results demonstrated that FPEs also prevented liver damage via their antioxidant properties (Figure 3).

### 3.7. Treatment with FPEs Reduced the Oxidative Stress Marker 8-oxo-dG

A part of mammary gland hyperplasia is the early pathological changes of breast cancer. Oxidative stress plays a crucial role in breast cancer induced by estrogen. The biomarker of 8-oxo-dG is one of the predominant forms of free radical-induced oxidative lesions and has therefore been widely used in the evaluation of oxidative DNA damage and carcinogenesis [23].

**Figure 4:**
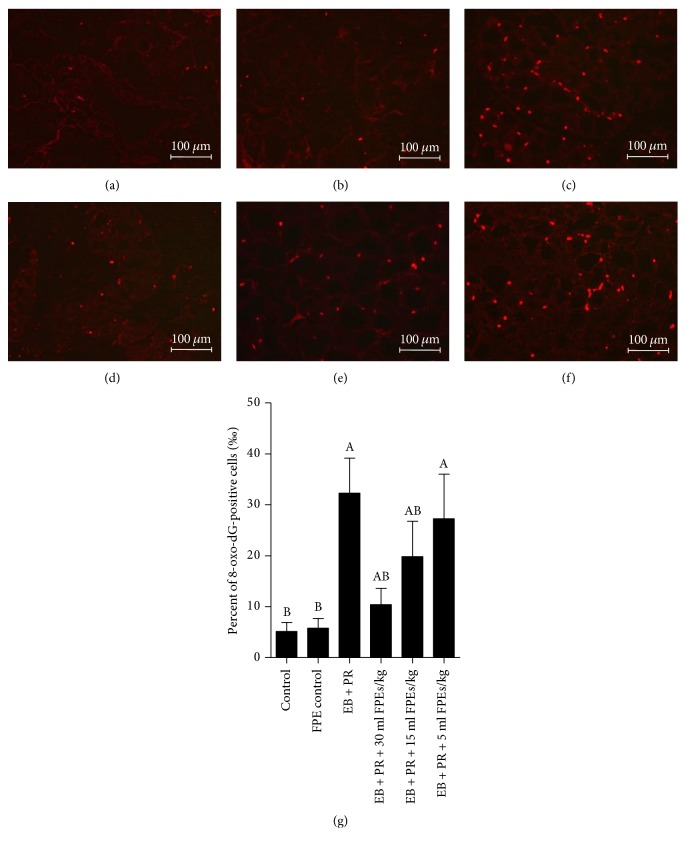
Effect of SPE supplementation on oxidative stress in mammary gland hyperplasia of rats induced by estradiol benzoate and progestin administration. (a–f) Representative images of 8-oxo-dG immunostaining in the mammary glands (200x). No apparent 8-oxo-dG-positive cells were observed in the control group (a) and the FPE-treatment-alone group (b). A high percentage of 8-oxo-dG-positive cells were observed in the estradiol benzoate and progestin treatment group (c). Scale bar, 100 *μ*m. However, the percentage of 8-oxo-dG-positive cells decreased upon treatment with FPEs at 30 ml/kg (d), 15 ml/kg (e) and 5 ml/kg (f). (g) Quantification of 8-oxo-dG-positive cells in the mammary glands by ELISA. Data are presented as mean ± SEM. ^A^*p* < 0.05 compared with the control group; ^B^*p* < 0.05 compared with the EB + PR group.

Results of IHC and ELISA analyses showed that the number of cells expressing 8-oxo-dG in mammary gland tissues was increased by estradiol benzoate and progestin. By contrast, the number of 8-oxo-dG-positive cells was remarkably reduced in the larger-dose and the middle-dose FPE treatments (compared with that in the model group, *p* < 0.05) (Figure 4).

**Figure 5:**
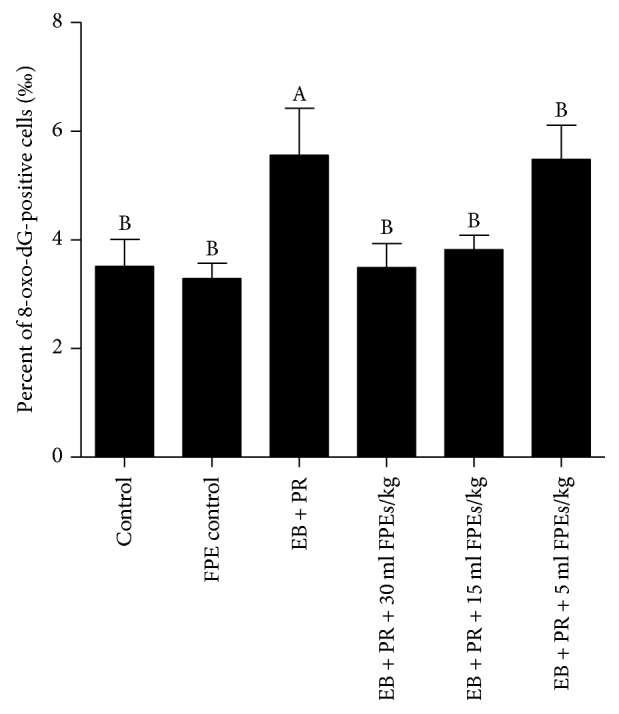
Quantification of 8-oxo-dG-positive cells in the liver by ELISA. Data are presented as mean ± SEM. ^A^*p* < 0.05 compared with the control group; ^B^*p* < 0.05 compared with the EB + PR group.

ELISA was conducted to detect 8-oxo-dG content in liver tissues. Similarly, the content of 8-oxo-dG in liver tissues of the animal models was reduced in the larger-dose and the middle-dose FPE treatments (compared with that in the model group, *p* < 0.05). The 8-oxo-dG contents in livers of rats administered with these two doses of FPEs had recovered to the level in the control group (Figure 5).

## 4. Discussion

The pathogenesis of mammary gland hyperplasia remains unclear, and studies suggest that this condition could be attributed to endocrine dyscrasia [24]. Increase in estrogen level in the body is known to promote multiplication of mammary gland cells, and long-term high-estrogen stimulation destroys the balance of mammary gland tissue hyperplasia and recovery during menstrual cycles. These phenomena result in gland duct expansion, adenomatosis, and acinus increase and further cause pathological mammary gland hyperplasia [20]. A large amount of reactive oxygen species (ROS) was found to be produced by long-term high-estrogen stimulation, which in turn caused oxidative stress injury in mammary gland tissues [25–27]. Given the aforementioned mechanism of mammary gland hyperplasia, estrogen and progestin can be utilized to induce mammary gland hyperplasia in model rats [18, 28]. In the present study, model rats with mammary gland hyperplasia induced by estradiol benzoate and progestin exhibited obvious elevation of estrogen level in the serum. Concomitant with this rise in estrogen level, severe pathological changes were observed. Oxidative stress is also a crucial factor in mammary gland hyperplasia. After mammary gland hyperplasia was induced by estrogen and progestin, SOD and GSH-Px activities, which are the major antioxidant enzymes that capture harmful active oxygen, in the serum and mammary glands of rats, were found to be obviously decreased.

FPPs are widely applied as functional food in the treatment and remission of various diseases [29, 30]. Although the mechanism underlying their therapeutic effects still remains unclear, antioxidant activity is one of its major functions [12, 31]. Antioxidant activity has been shown to be effective in the treatment and remission of complications related to age [32], erythrocyte oxidative damage [13], wound healing in diabetics [11, 33, 34], and AD [14]. In this study, FPEs can not only improve oxidative indices in the animal models, but can also increase SOD activity in the normal animals. This indicates that FPEs exhibit strong antioxidant activity, and their protective effect on mammary gland hyperplasia is likely related to this property.

Estrogen can induce cell multiplication and inhibit DNA repair [35, 36]. Previous studies showed that breast tumor is induced by estrogen through oxidative DNA damage [25, 37, 38]. The biomarker 8-oxo-dG is a product of ROS that induces oxidative DNA damage, which is regarded as a cell marker of oxidative stress and oxidative DNA damage, and is the most common product of oxidative stress damage induced by estrogen [39, 40]. After long-term estrogen inducement, 8-oxo-dG-positive cells in the mammary gland obviously increased, but improved when the animal models were given with FPEs. This finding indicates that, in addition to its antioxidant activity, FPEs also have the ability to inhibit oxidative DNA damage. This is consistent with a previous report that FPP can reduce oxidative DNA damage and improve cytokine balance [41].

In this study, a panel of blood biochemical markers in the experimental animals were detected. The AST, ALT, and TBIL indices in the serum of the animals with mammary gland hyperplasia induced by estrogen were remarkably increased. Meanwhile, these biochemical indices in the animal models treated with FPEs had recovered to the level of the control group. This result indicates that estrogen can cause mammary gland hyperplasia and damage other visceral organs. However, FPEs can protect these organs from being injured. AST and ALT are markers of heart and liver damage, whereas TBIL is a marker of liver damage. After pathological detection of the major visceral organs, no obvious pathological changes to the heart, spleen, lung, kidney, ovary, and uterus were observed. However, pathological changes in the liver, which mainly consisted of inflammatory cell infiltration, were still induced. However, high-dose FPEs completely protected the liver from being injured. Oxidant indices and oxidative DNA damage markers indicate that FPE acts as an antioxidant to protect the liver from damage. Previous studies showed that FPP can protect liver activity and inhibit liver cancer, which is mainly achieved via its antioxidant activity [42, 43]. Oxidative stress plays an important role in mediating hepatotoxicity associated with many diseases [44, 45]. FPEs improve and protect the organs, and in turn improve blood biochemical indexes, through their antioxidative effects.

FPP is available in the market in powdered form after papaya fermentation with edible fungi. FPEs, the extracting solution fermented by papaya through the collaboration of *A. oryzae* and yeasts for six months, can be easily prepared and consumed. Clinically, FPEs are mainly used for improving digestion and immune regulation, endocrine dyscrasia improvement, and weight reduction. In this study, three FPE treatment groups (each receiving 30, 15, or 5 ml/kg FPEs) was used for evaluating the protective effect on mammary gland hyperplasia. The results provide convincing evidence that FPEs with higher and middle doses could obviously improve hormone levels, clinical signs, oxidative indices, biochemical index, and pathological changes in model animals of mammary gland hyperplasia, and with smaller dose had no significant modulatory role. Moreover, the higher dose is the optimal dose, which was converted into an adult dose 70 ml/kg approximately according to body surface area. In general, the clinical dose of adult women is 100 ml/d. A 1-month safety evaluation of FPEs for SD rats was conducted. The results showed that blood biochemistry, hematology, histopathology, ingestion, and water intake of the rats administered the recommended clinical dose of FPEs four times did not obviously change. However, an obvious decrease in body weight was documented, which is also a possible reason for the observed change in body shape.

Studies have shown that mammary gland hyperplasia induced by estrogen can be effectively inhibited by FPEs via their antioxidant effects. In addition, FPEs can also improve and repair oxidative damage in the liver. FPEs contain abundant nutrients, such as alpha-tocopherol [8], flavonoid [9], glutamic acid, cystine, cytosine, nicotinic acid, pipecolic acid, homoserine, quinic acid, and glucuronic acid, which possess antioxidant and antitumor functions. In recent years, liquor obtained via fruit fermentation has become popular in China and is commonly sold as commercial food. Each active element in FPEs should be further analyzed. Future studies should focus on the functions and mechanism of FPEs in various diseases and in developing FPEs as functional food.

## Figures and Tables

**Table 1:** Effect of FPEs on the nipple and sex hormones in rats.

Groups	Nipple (mm)		Sex hormones			
	Height	Diameter	E2 (pg/ml)	P (pg/ml)	LH (pg/ml)	FSH (pg/ml)
Control	1.22 ± 0.14^b^	1.27 ± 0.10^b^	33.56 ± 12.43^b^	70.44 ± 13.34^b^	4.63 ± 0.69^b^	5.17 ± 0.83^b^
SPE control	1.27 ± 0.11^b^	1.29 ± 0.14^b^	38.24 ± 8.31^b^	79.25 ± 16.08^b^	5.27 ± 1.08^b^	4.63 ± 1.32^b^
EB + PR	1.86 ± 0.17^a^	1.78 ± 0.12^a^	166.55 ± 46.51^a^	151.22 ± 26.25^a^	9.16 ± 2.71^a^	18.46 ± 3.15^a^
EB + PR+ 30 ml SPE/kg	1.45 ± 0.23^b^	1.41 ± 0.21^b^	62.55 ± 42.63^a,b^	125.53 ± 19.23^a,b^	5.77 ± 1.54^b^	8.64 ± 3.24^b^
EB + PR+ 15 ml SPE/kg	1.64 ± 0.24^a^	1.46 ± 0.18^ab^	98.24 ± 31.31^ab^	142.65 ± 7.19^a^	7.15 ± 2.26^ab^	10.52 ± 2.54^ab^
EB + PR+ 5 ml SPE/kg	1.76 ± 0.09^a^	1.72 ± 0.16^a^	135.62 ± 36.89^ab^	138.97 ± 16.52^a^	8.23 ± 1.84^a^	16.63 ± 2.11^ab^

SPE: fermented papaya extract; EB: estradiol benzoate; PR: progestin; E2: estradiol; P: progesterone; LH: luteinizing hormone; FSH: follicle-stimulating hormone. Statistical differences are within the individuals at the same column. ^a^*p* < 0.05 compared with control group; ^b^*p* < 0.05 compared with EB + PR group.

**Table 2:** Effect of SM on estradiol benzoate and progestin induced mammary gland changes on histopathology.

Groups	Mammary gland hyperplasia	Lobule increase	Acinar increase	Mammary duct and lumen ectasia	Mammary duct and lumen secretion
Control	−	−	−	−	−
FPEs control	−	−	−	−	−
EB + PR	+++	+++	+++	+++	+++
EB + PR+ 30 ml FPEs/kg	+	+	+	++	+/−
EB + PR+ 15 ml FPEs/kg	++	++	++	+++	+
EB + PR+ 5 ml FPEs/kg	+++	+++	+++	+++	++

FPEs: fermented papaya extracts; EB: estradiol benzoate; PR: progestin. The histopathology changes were determined at the end of experiment. −: none; +/−: some have and some not; +: mild; ++: moderate; +++: severe.
